# Computing Platforms for Big Biological Data Analytics: Perspectives and Challenges

**DOI:** 10.1016/j.csbj.2017.07.004

**Published:** 2017-08-14

**Authors:** Zekun Yin, Haidong Lan, Guangming Tan, Mian Lu, Athanasios V. Vasilakos, Weiguo Liu

**Affiliations:** a Shandong University, Jinan, Shandong, China; b Institute of Computing Technology, Chinese Academy of Sciences, China; c Huawei Singapore Research Centre, Singapore; d Department of Computer Science, Electrical and Space Engineering, Luleå University of Technology, Skellefteå SE-931 87, Sweden

**Keywords:** Computational biology applications, Computing platforms, Big biological data, NGS, GPU, Intel MIC

## Abstract

The last decade has witnessed an explosion in the amount of available biological sequence data, due to the rapid progress of high-throughput sequencing projects. However, the biological data amount is becoming so great that traditional data analysis platforms and methods can no longer meet the need to rapidly perform data analysis tasks in life sciences. As a result, both biologists and computer scientists are facing the challenge of gaining a profound insight into the deepest biological functions from big biological data. This in turn requires massive computational resources. Therefore, high performance computing (HPC) platforms are highly needed as well as efficient and scalable algorithms that can take advantage of these platforms. In this paper, we survey the state-of-the-art HPC platforms for big biological data analytics. We first list the characteristics of big biological data and popular computing platforms. Then we provide a taxonomy of different biological data analysis applications and a survey of the way they have been mapped onto various computing platforms. After that, we present a case study to compare the efficiency of different computing platforms for handling the classical biological sequence alignment problem. At last we discuss the open issues in big biological data analytics.

## 1 Introduction

Biological sequence data are growing exponentially. The rate of growth over the last decade has also been truly astonishing, with the total amount of sequence data produced doubling approximately every seven months [1]. Data growth rate will continue for the foreseeable future, since multiple concurrent genome sequencing projects have begun, with more to come. The availability of big biological data is vital for evolutionary studies. For the first time we can study the governing factors in the evolutional processes of whole genomes. This is therefore an exciting era for evolutional biology. However, as the semi-conductor lithography process approaching its physical limits, the growth of transistors on a single chip is much slower than the growing rate of biological sequence data. The computational load is further compounded by the addition of new data sources (many completed genomes are being reported monthly), increase in the size and number of queries, a growing user base of bioinformatics scientists, new algorithms and methods of analysis. Because of the following factors, it usually takes long runtimes to solve big biological data analysis problems:

• Sequencing technologies to produce biological data are prone to errors. Thus high complexities will be introduced into algorithms in order to handle these errors and uncertainties.
• Big biological data analysis problems have a very high computational requirements even the corresponding algorithms have polynomial time complexities [2].
• Due to inherent algorithmic complexities, many biological data analysis problems are both data-intensive and compute-intensive. HPC may provide an efficient tool to solve these problems.

This is a new area of biological sciences where computational methods are essential for the progress of the experimental science, and where algorithms and experimental techniques are being developed side by side.

Traditionally, HPC platforms such as supercomputers were rare and available for only the most critical problems. Since the mid-1990s, however, the availability of supercomputers has changed dramatically. With multi-threading support built into microprocessors and the emergence of multiple processor cores on a single silicon die, supercomputers are becoming ubiquitous. Now, almost all university computer science department has their own HPC platforms. Given the exponential growth in the size of biological sequence data, the computational biology (CB) area has taken dramatic leaps forward with the availability of computational resources. Traditional uses of HPC platforms in scientific computing usually involve problems described in structured grids, with well-defined regular data structures. In contrast, many problems in CB have irregular structures, which appears to be significantly more challenging to parallelize. Thus, the effective use of HPC platforms will become increasingly important in CB. This continues to remain a largely unexplored territory, and is the principal motivation behind our survey work.

In the past few years, the fast increasing power of new generation many-core architectures has opened up a range of new possibilities to achieve HPC for a variety of applications. Graphics Processing Units (GPUs) are one of the most widely used general-purpose many-core architectures. These commodity chips have enhanced their programmability to perform more general computational tasks than the graphics processing they were originally designed for. Examples include scientific computing [3], image processing [4], computational biology [5], electronic design automation (EDA) [6] and data science [7], etc. The computer video game market have driven the evolution of GPUs to yield relatively cheaper price per unit and very rapid iteration of hardware architectures. Intel Xeon Phi is another popular many-core architecture. It is based on the Intel's Many Integrated Core (MIC) architecture which integrates much more simplified hardware cores compared to traditional CPUs. With the easy programmability of x86-based Xeon Phi, these chips are now widely used. Scientists and engineers in a variety of fields have presented their design and implementation of parallel algorithms on Xeon Phi. Examples include scientific computing [8], database operations [9] and computational biology [10]. Limited by power consumption and advances in lithography, the many-core architectures shows better power-efficiency than the traditional multi-core CPUs. Thus, the many-core based platforms are even more attractive for the HPC community in the near future. However, there are still many challenges to be solved for the CB scientists to facilitate efficient usage of many-core based HPC platforms. In this paper, a survey and taxonomy of HPC big biological data analysis applications on various computing platforms are presented.

The rest of this paper is organized as follows: in Section 2 we present the characteristics of big biological data and popular computing platforms. In Section 3, we provide a taxonomy of different biological data analysis applications and how they have been mapped onto various computing platforms. Section 4 presents a case study to compare the efficiency of different computing platforms for handling the classical biological sequence alignment problem. Then we discuss the open issues in big biological data analytics in Section 5. Finally, Section 6 concludes this paper.

## 2 Big Biological Data and Computing Platforms

In this section, we first talk about the characteristics of big biological data. Then we introduce popular computing platforms used in practice, and the corresponding programming models.

### 2.1 Characteristics of Big Biological Data Analytics

Over the past decades, whole genome sequencing (WGS) technologies are rapidly progressing. Nowadays, human genomes can be sequenced around 50,000 times faster than that in 2000 [11], but with the cost of only 1/25,000 [12]. With this exponential growth of sequence data, rich biological data analytics applications are developed and studied, such as sequence alignment (including short read alignment), genome assembly, single nucleotide polymorphism (SNP) detection, and genome-wide association study (GWAS). Particularly, many of such applications share a few common characteristics. Understanding those characteristics thoroughly first is helpful to identify the challenges for computational science. We summarize three major characteristics as follows: huge volume of data, extremely long running time and application dependency.

#### 2.1.1 Huge Amount of Data

As the sequencing speed has been greatly improved but with significantly reduced economic cost, huge amount of sequence data is generated everyday in sequencing centers. For example, a modern Illumina sequencing machine is able to generate over 1.8 terabases of data per week [13]. As a result, in a typical sequencing center, hundreds of TB of sequence data is produced per day. Such high pressure of data volume not only introduces challenges to hardware support, but also to computational scientists to process data efficiently and effectively.

#### 2.1.2 Extremely Long Running Time

A biological data analytics application may run for days or even months because of two reasons. First, the large amount of sequence data requires high throughput of data processing. For example, short read sequence alignment tools are used by scientists everyday to process sequence data. Though the algorithm has relatively low time complexity by employing advanced indexing techniques [14], [15], the alignment task still has to take long time to process all data. Second, some applications have extremely long running time because of large data size as well as high computation complexity. For example, state-of-the-art genome assembly tool SOAPdenovo2 [16], has to take a few days with the consumption of hundreds of GB of memory to finish the construction for a single human's genome. Other applications such as SNP detection [17] and GWAS [18] may also take days or even months to finish processing one dataset.

#### 2.1.3 Application Dependency

In a sequencing center, different data analytics tools are typically developed individually but used together in workflows as components. In a representative workflow, sequence data is first produced by sequencing machines, and then aligned to a reference sequence using short read alignment tools. Then the alignment results are sorted using an external sorting program. Next, the sorted alignments are fed into a SNP detection program. The result of SNP detection may be further as input for other GWAS applications, such as SFS estimation [18]. Because all these programs are developed separately, both the input and output data are stored on disks. As a result, when a workflow consisting of different data analytics tools, it introduces significant performance overhead from disk I/O due to data movement.

Those three major characteristics introduce corresponding challenges for efficient, scalable and productive biological data analytics. Researchers have invested huge efforts into developing efficient and effective biological data analytics tools.

### 2.2 Computing Platforms and Programming Models

For the last decades, we have witnessed abundance of computing platform choices for analyzing biological data. The choices provide a number of options to obtain efficiency gain or the capability to implement biological data analysis algorithms. These options include general-purpose platforms like multicore parallelism, high-performance computing clusters and cloud computing, and accelerators like GPUs (Graphics Processing Units), Intel Xeon Phi and FPGA (Field-programmable Gate Array). Aside from the multiple platform options, there exists a variety of programming models in which algorithms can be implemented. Programming model choices tend to be particularly diverse due the extra consideration of performance and productivity. Currently, people have put efforts on programming biological data analysis programs with mainstream programming models including OpenMP, CUDA/OpenCL, message passing (MPI) and map-reduce (Hadoop, SPARK), which are adopted to exploit the diversity of parallelism on computing platforms. The wide range of architectures and programming models presents both opportunities and challenges for biological data analysis scientists and engineers. Fully exploiting the available hardware resources requires adapting some algorithms and redesigning others to enable their concurrent execution.

## 3 Taxonomy

In this section, we preset a taxonomy of the different biological algorithms that have been implemented on different platforms. We categorize them into two main groups: biological algorithms for whole sequences and biological algorithms for NGS. For each category, we choose a series of classic algorithms to discuss their implementations on multi-core, GPU, MIC, cluster and cloud. We mainly focus on the optimization skills on different platforms, like memory access pattern, computation density and I/O density, see Table 1.

**Table 1:** Classic bioinformatics applications. C&C is short for cluster and cloud. In this table, at first we give a short description about specifications of each application. Then three major characteristics are listed: memory access pattern, computation density and I/O density. At last we list platforms the applications have been implemented on.

Application	Specification	Memory access pattern	Computation	I/O	Platform			
					Multi-core	GPU	MIC	C&C
ClustalW	Classic but old	Regular and irregular	High	Low	[19]	[20]	[21]	[22]
Clustal Omega	Fast and scalable	Regular and irregular	High	Moderate	[23]	NA	NA	NA
Smith-Waterman	Small database, optimal results	Regular	High	Moderate	[24], [25]	[26], [27]	[28], [28], [29]	[25]
Blast	Large DB, heuristic algorithm	Irregular and regular	High	Low	[30], [31]	[5], [32], [33]	Yes	NA
BLAT	In memory, fast than Blast	Regular and irregular	High	Moderate	NA	NA	NA	NA
Bowtie2	Typical short read alignment tool	Irregular	Moderate	Moderate	[34]	NA	NA	NA
BWA	Typical short read alignment tool	Irregular suffix array	Low	High	[35]	NA	[36]	[37]
mrFast	Short read, all mapper	Regular filter strategy	High	Low	[38]	NA	NA	NA
SPADES	Fast assembler, single and multi cell	Irregular	Low	High	[39]	NA	NA	NA
BFCounter	Error correction	Regular	High	Low	[40]	NA	[41]	NA
Fiona	Error correction	Irregular	Low	High	[42]	NA	NA	NA

At first we list some parallel applications about parallel algorithm design and optimization techniques, see Tables 2 and 3. Two of listed applications are implemented on Intel MIC platforms including XSW and LSDBS. Four are implemented utilizing NVIDIA CUDA and FHAST is designed for FPGA heterogeneous computing. The rest are designed for multi-core platform. We notice that all of these applications are parallelized in coarse-grained way, and in order to exploiting the high computing performance of GPU and MIC fine-grained parallel strategies are usually used. SIMT and SIMD are two most popular techniques for fine-grained parallelism. SIMT(single instruction multiple thread) is an execution model used in GPUs, in NVIDIA GPU threads in one warp execute concurrently using a single instruction. SIMD(single instruction multiple data) describes the VPUs could operate multiple data (a vector) with a single instruction. For most algorithms with regular memory access pattern, using fine-gained SIMT on GPUs and SIMD on multi-cores makes applications several times faster.

**Table 2:** Parallel algorithm design.

Application	Description	Data organization	Coarse-grained parallel	Fine-grained parallel
SWIPE [25]	Multi-core Smith-Waterman database search	Sequence profile	Multi-thread	SIMD
XSW [29]	Smith-Waterman database search on Xeon Phi	Pre-processing	Multi-thread	SIMD
CUDASW++ [43]	Smith-Waterman database search on GPUs	Texture filter	Data	SIMT
LSDBS [9]	Large-scale database search on Xeon Phi	Pre-processing	Multi-thread	SIMD
CUDA-BLASTP [5]	Accelerating BLASTP utilizing CUDA	DFA reorganization	Data	SIMT
MSA-CUDA [20]	ClustalW accelerated using CUDA	Sorting	Data	SIMT
FHAST [44]	FPGA-based acceleration of BOWTIE in hardware	Index	Data	–
BWA [45]	A typical best mapper algorithm	BWT & FM-index	Multi-thread	–
BitMapper [46]	A typical all mapper algorithm	Hash index	Multi-thread	SIMD
DecGPU [47]	GPU based error correction algorithm	bloom filter	Data	SIMT

**Table 3:** Application optimization.

Application	Data transfer	Memory access	Cache	Load balance	Heterogeneous computing
SWIPE	Synchronized	Score profile	–	Dynamic	CPU
XSW	Asynchronized	Score profile	–	Dynamic	Xeon Phi native
CUDASW++	Synchronized	Query profile	Texture	Static	CPU + GPU
LSDBS	Asynchronized	Score profile	Multi-pass	Dynamic	CPU + Xeon Phi
CUDA-BLASTP	Synchronized	Memory coalescing	DFA index table	Static	GPU
MSA-CUDA	Synchronized	Memory coalescing	–	–	GPU
FHAST	Synchronized	–	–	–	FPGA
BWA	–	Index	–	–	–
BitMapper	–	–	–	Dynamic	CPU
DecGPU	Asynchronized	Memory coalescing	–	Dynamic	GPU

### 3.1 Whole Genome Sequence

#### 3.1.1 Dynamic Programming (DP) Algorithms

##### 3.1.1.1 Smith-Waterman Algorithm

Smith-Waterman algorithm, first proposed by Temple F. Smith and Michael S. Waterman in 1981 [48], is a classical sequence alignment algorithm. It performs optimal local sequence alignment between two nucleotide sequences or protein sequences. Smith-Waterman algorithm adopts the dynamic programming strategy, hence, the algorithm guarantees to find the optimal alignment with respect to the scoring system. However, the quadratic time and space complexity limits its efficiency for database search problem. A linear space approach was proposed by Miller Webb and Myers Eugene in 1988 [49], which is the very basics of modern implementations. Efforts on accelerating Smith-Waterman algorithms have primarily involved appeals to hardware parallelization. For CPU approaches, SIMD instruction sets are used to invoke data parallelism. Early approaches [50], [51] focus on finding inherent parallelism in the algorithm. The wavefront method takes advantage of the fact that matrix cells on the same anti-diagonal are independent. The major shortcoming is that the SIMD vectors are not fully filled at startup and finishing stages. A pretty-fast SSE2 approach proposed by Farrar in 2007 [24] uses a striped strategy to overcome the dependency along the query sequence. Rognes proposed SWIPE [25] in 2011, which is considered as the fastest SSE implementation. Unlike previous approaches, SWIPE takes inter-sequence parallelism and the score profile strategy for efficient score fetching. This is the first time that Smith-Waterman implementations achieve BLAST-level performance with respect to specific score matrix. Rucci et al. [52] propose SWIMM in 2015 to take advantage of the novel AVX2 instruction set. Benefited from wider vector processing capabilities, the authors report a performance of 354.8 GCUPS on dual 14-core Intel Xeon CPUs, outperforms the SWIPE by a factor of 1.5.

On GPUs, Liu et al. [53] first proposed a streaming approach in 2007, which is considered as the first effective GPGPU implementation. There are various implementations on Nvidia's GPUs, of which the best is CUDASW++ [26], [27], [43]. This work removed query length limitations which is often required by mapping the problem set onto a texture. With the 8-bit video SIMD instruction introduced in the Kepler architecture, the 3.1 version of CUDASW++ achieves over 130 GCUPS on a single Nvidia Tesla K40c, which is at least 3 × faster than the 8-core CPUs without AVX2 support. More over, the CUDASW++ 3.1 could cooperate CPUs and GPUs to work together to fully utilize the computing power available in the system.

On Intel Xeon Phi computing platform, XSW [29] and SWAPHI [28] are the first works to report the performance at 62 GCUPS and 70 GCUPS, respectively. The original XSW implementation is based on native model, which limited the database size. In the follow up work LSDBS [9] proposed in 2015, the limitation on database size is removed, and the CPUs are also involved in the computing pipeline. LSDBD uses a dynamic distribution strategy to balance the workload among the CPUs and Xeon Phi cards, which is proved to be effective and scalable. SWIMM also proposed a Xeon Phi implementation based on guided auto-vectorization with the performance at 41 GCUPS.

##### 3.1.1.2 ClustalW

ClustalW [54] is a famous progressive algorithm for multiple sequence alignment. Since it first introduced in 1990s, ClustalW has been widely accepted by biologists as a fast and accurate MSA tool. ClustalW has been implemented on different platforms, MT-ClustalW [19] on multicore platform, streaming algorithm on early GPGPUs [53], CUDA-MSA [20] and GPU-ClustalW [55] on GPUs utlizing CUDA, a simple implementation on Xeon Phi [21] and ClustalW-MPI [22] on CPU clusters. ClustalW consists of three main stages: pairwise distance computation, guide tree construction and profile-profile alignment along the guide tree.

Most works on HPC platforms pay much attention to stage one for it's the most time consuming part with the time complexity *O*(*N*^2^*L*^2^). Li presents ClustalW-MPI implemented using MPI which is targeted for clusters. But ClustalW-MPI only parallelize the first and the third stages of ClustalW using coarse-grained parallel strategies. MT-ClustalW is designed for multi-core processors but merely parallelizes stage 2 using Pthreads library on the basis of ClustalW-SMP [56]. MSA-CUDA is the first known ClustalW implementation on GPU using CUDA, and it parallelize all three stages of the progressive alignment alignment. In MSA-CUDA, Liu describe a novel algorithm to reconstruct the guide tree in parallel. But MSA-CUDA doesn’t supply large scale dataset (MSA-CUDA crashes when running 8000 sequences as input with average length 1000 bp). CUDA-ClustalW is a recently presented version of ClustalW on GPU. CUDA-ClustalW follows similar strategies as MSA-CUDA but CUDA-ClustalW supports multiGPUs which means it can handle larger dataset than MSA-CUDA. In 2014 Borovska et al. [21] give a discussion on using Intel Xeon Phi to accelerate ClustalW, they try to use MPI and OpenMP hybrid programming to map ClustalW on Intel Xeon Phi. Their performance estimation and the analyses show that the hybrid parallel program implementation utilizing MPI and OpenMP of ClustalW scales well as the number of cores increase up to 60 cores.

#### 3.1.2 Heuristic Algorithms

##### 3.1.2.1 Blast (Basic Local Alignment Search Tool)

Blast [30] is one of the most common used biology gene sequence database search tools, and it can search proteins and nucleic acids gene database. After it was proposed last century, that article has been cited over 50000 times. It is a heuristic algorithm, which is different from classical dynamic programming algorithm (Smith-Waterman). Blast is faster but the precision of result is lower than dynamic programming algorithm. With the development of HPC, many parallel research about Blast has been done, such as NCBI-Blast, FSA-Blast [31], CUDA-Blastp [57], cuBlastp [57] and Hadoop-Blast [58]. NCBI-Blast is the most popular blast implementation, which is on multi-core platform, and is supported by NCBI. Hadoop-Blast implements a distributed BLASTP by combining Hadoop and multi-GPUs, and it achieves better availability and fault tolerance.

Blast algorithm can be divided into four stages. FSA-Blast algorithm optimize the first stage of blast. It uses a deterministic finite automaton (DFA) model to optimize the cache hit rate. The ordinary hit lookup table is the simple one-dimensional array. Cache hit rate can be optimized by utilizing DFA model because it organizes the data in neighbor location that will be accessed in the near future. This optimization has become a basic part of many other blast algorithm.

CUDA-Blastp algorithm add a extra filter in traditional blast algorithm to filter most apparently wrong results, and retain the similar results. Coarse-grained parallelism is thread level data parallel in GPU. The fine-grained parallel uses the classical wave-front Smith-Waterman parallel algorithm.

CuBlastp algorithm is another GPU implementation which optimizes the first two stages of blast algorithm. The irregular memory access pattern of blast algorithm is difficulty in first two stages. To our knowledge, the cuBlastp is the first fine-grained parallel implementation of the first two stages of blast algorithm.

#### 3.1.3 Hidden Makrov Model (HMM) Based Algorithm

##### 3.1.3.1 HMMER

[59] is another commonly used biological sequence database search tool which was first introduced in 1998. It does this by comparing a profile-HMM to database sequences. The profile-HMM is constructed by using hmmbuild program in HMMER package. HMMER3 [60] is totally rewrite from the HMMER in order to get better performance by using a heuristic filter to find high-scoring un-gapped matches. HMMER3 also support multi-thread in coarse-grained parallelism and SIMD in fine-grained parallelism. Both the heuristic filter and the parallel scheme make HMMER3 much faster than the old version of HMMER.

Moreover, in recent years in order to take advantages of new high performance hardwares, several works on accelerating HMMER on GPUs have been reported such as [61] in 2010 and [62] in 2012. And Oliver et al. [63] reported their work on accelerating HMMER searching using FPGAs in 2008. As far as we know, there is not HMMER implementation on Intel MIC platform reported yet.

### 3.2 Next Generation Sequence (NGS)

#### 3.2.1 Mapper

##### 3.2.1.1 BWA

BWA is a famous algorithm for NGS read alignment. It is introduced in recent years, and has three algorithms, BWA-aln [35] for short reads, BWA-sw [45] for long reads, and BWA-mem [64] which is suitable for both short reads and long reads. BWA has been widely accepted by biologists as an accurate NGS read alignment tool. BWA has been implemented on regular multicore platforms, and there is also a pBWA [65] for clusters. A CUDA-based project, CUSHAW [66] has similar functions with BWA.

BWA is one of the best-mappers, which means finding a best mapping position on a reference sequence of each input read. It mainly contain the following stages:

• Build a FM-index for reference sequence.
• Search for patterns of a read in the index of reference, find some mapping position.
• Detailed alignment and generate the alignment information.

The key data structure in the BWA algorithm is FM-index, it is one kind of full text index based on BWT (Burrows-Wheeler transform). Searching a pattern with length *n* in the FM-index of a reference has a time complexity *O*(*n*), but this procedure comes with badly irregular memory accesses. This procedure is one of the most time-consuming parts of the BWA algorithm, so making full use of SIMD instructions for fine-grained parallelization in BWA algorithm or migrating BWA algorithm is a very hard task.

The only well-known and similar approach on heterogeneous devices is CUSHAW, which stores the index in global cached memory of GPUs, but the pseudocode of its CUDA kernel shows it uses an algorithm like BWA to do searching in BWT, with some discrete global memory access in the kernel, which is not able to make full use of the power of GPUs. And as its performance evaluation shows it doesn’t achieve a landslide win on performance when the length of reads grows to 100bp compared to CPU implementations.

In the coarse-grained parallelization, BWA originally uses a multi-threading strategy in a single node, it divides tasks to blocks, and dispatch threads for each block with static load balancing in each block.

##### 3.2.1.2 All Mapper

All mapper is desirable in many applications such as ChIP-seq experiments [67] and RNA-seq transcript abundance quantification [68], for it can identify all candidate locations. To our best knowledge, all existing approaches of all mapper are based on seed-and-extend paradigm and runs on CPU. mrFAST [38] is one of the popular seed-and-extend based mappers. It first builds a hash index for reference genome and then takes use of the hash index to retrieve all candidate locations for each read to verify. Recently, mrFAST incorporates FastHash [69] to filter clearly false mappings before verification. mrFAST does not support multi-threading, which means it will take a long mapping time when dataset is large.

As for coarse-grained parallelization, RazerS3 [70] has developed a load balancing scheme. RazerS3 has implemented a pigeonhole filter, which means it takes much less time to filter less false locations. Since time spent for verification dominates the whole running time and the verification can be done dynamically, all threads can finish almost simultaneously.

Hobbes [71] uses a dynamic programming algorithm to choose *k* + 1 non-overlapping *q*-grams with lowest frequency, where *q*-grams are substrings of length *q*. Thus, the number of candidate locations is minimal. Hobbes 2 [72] selects *k* + 2 *q*-grams instead of *k* + 1 and only verifies locations that appear at least twice to filter more false candidates. Hobbes and Hobbes 2 also create extra two threads which are corresponding for input reads and output results. Therefore, memory consumption of Hobbes and Hobbes 2 will not be affected by the number of reads or the number of mappings.

Both RazerS3 and Hobbes 2 adopt a banded Myers algorithm [73] to verify each candidates one by one after filtration. To further investigate fine-grained parallelism, BitMapper [46] extends the banded Myers algorithm to verify multiple candidates against a read simultaneously by loading several bit vectors into a machine word. Moreover, it has implemented this refined algorithm with 128-bit registers and SSE/SSE2 instruction set on CPU, which significant reduces verification time. The 512-bit VPU of Xeon Phi coprocessor is usually suitable to vectorized and accelerate bit-parallel algorithms such as Wu-Manber approximate pattern matching algorithm [74].

#### 3.2.2 Error Correction

##### 3.2.2.1 Error Correction

The Next Generation Sequencing (NGS) produces massive amounts of reads that contains far more errors than traditional sequencing methods. A number of methods have been developed to prune such errors. These error-correction methods could be categorized into three types: (i) k-spectrum based, (ii) suffix tree/array-based and (iii) MSA-based methods. The k-spectrum based methods decompose reads into a set of all the k-mer segments that appears in them. The k-mers that belong to the same genomic location tends to be within a small Hamming distance from each other, which provides a method to directly align sequences by identifying such a k-mer set without resorting to the time-consuming MSA. Errors can be corrected by converting each constituent k-mer to the consensus. The suffix tree/array based error-correction methods are generalization of the k-mer-based approach. They handle multiple k values and their corresponding threshold. The MSA-based methods first use the MSA tools to generate the alignment. Corrections are applied when the reads involved in the same alignment appears at a moderate number, and the maximal edit distance between the constituent reads and the consensus of the alignment is blow an user-defined threshold [75]. Many techniques for error correction have been developed in recent years. The BLESS [76] is a distributed k-mer spectrum-based error-correction tool. It adopts a Bloom filter with the ability to tolerate a higher false-positive rate. The CUDA-EC [77] is a scalable parallel algorithm for correcting sequencing errors in high-throughput short read data. It is a spectral alignment method developed for CUDA-enabled GPUs. The DecGPU [47] presents a distributed GPU-enabled error correction method for high-throughput short reads by combining CUDA and MPI. It features the capability to invoke the computing power of GPU clusters.

## 4 Case Study

The Smith-Waterman algorithm performs exhaustive search to find the optimal alignment between two biological sequences. The dynamic programming scheme guarantees to find the optimal result, but is computing demanding as well. The heuristic alternatives, such as the BLAST and FASTA, has been among the most influential biological tools. However, the heuristic scheme trades speed with sensitivity, which makes acceleration for Smith-Waterman algorithm still meaningful. Our motivation is to compare and find the best parallelization method with respect to hardware architectures. The platforms involve GPU and Intel MIC.

### 4.1 GPU

On GPUs, Liu et al. [53] first proposed a streaming approach in 2007, which is considered as the first effective GPGPU implementation. This work adopts the wavefront method. The problem is mapped as a graph problem to be solved by OpenGL APIs. As Nvidia announced their CUDA computing platform, general-purpose computing on GPUs becomes easy. Various implementations emerged of which the CUDASW++ series [26], [27], [43] is among the bests.

The first version of CUDASW++ is implemented for the first generation of Nvidia Tesla GPUs. This study implements intra-sequence parallelism and inter-sequence parallelism to find that the inter-sequence parallelism achieves better performance. The speedup over CPUs of the same generation using Farrar's method is not significant.

The CUDASW++2.0, which is optimized for the Fermi architecture, is a great success. The authors implemented the wavefront method, Farrar's vectorization method, and a novel SIMT method on CUDA-enabled GPUs. The wavefront and vectorization methods take intra-sequence parallelism, while the SIMT method adopts the inter-sequence parallelism. Unlike SWIPE, the CUDASW++2.0 uses query profile for efficient substitution score fetching. Texture memory is used to accelerate access to query profile and the subject sequences. In fact, the texture units on GPU can cover the overhead in assigning the scores to the correct thread, which is the major bottleneck for query profiles.

CUDASW++3.0 is considered as the state-of-art GPU implementation. It aligns CPUs together in the searching procedure. On the GPU side, the novel video SIMD instructions are adopted with inter-sequence parallelism. In order to further improve efficiency, the authors proposed a variant of query profile to reduce the shifting operations. The variant query profile achieves better performance, but meet a cache-miss problem with long query sequence whereby the L2-cache fails to hold the profile. The authors turns to use the standard query profile for long queries. On the CPU side, the SWIPE program is invoked for calculation. This study makes a static partition of the database to distribute workload to CPUs and GPUs. The ratio is defined over core number, clock speed and a tuning constant. There's a load-balancing problem when the tuning constant is not proper tuned. However, this constant is inconsistent with different hardware configurations.

### 4.2 Intel MIC

The recently released Xeon Phi coprocessor is based on the Intel Many Integrated Core (MIC) architecture. It offers many cores on a single die. Each core is designed to be power efficient while providing a high throughput for highly parallel workloads. A closer look reveals that the core uses a short pipeline and is capable of supporting 4 threads in hardware. There are 32 vector processing units (VPU) on each core. VPU is an important component of Xeon Phi and it features a novel 512-bit SIMD instruction set. Thus, the VPU can execute 16 single-precision or 8 double-precision floating operations per cycle in parallel. Intel has implemented a high bandwidth memory hierarchy on Xeon Phi. In this hierarchy, each core is equipped with a 32 KB L1 instruction cache, a 32 KB L1 data cache and a 512 KB unified L2 cache. The coprocessor could work in native mode, offload mode and symmetric mode. The native model uses the coprocessor as a standalone subsystem. The user needs to log on to the coprocessor like a remote host to carry out search tasks. The offload model works like GPUs to perform the computing-intensive tasks. The symmetric mode let the host CPU and the Xeon Phi coprocessor run in parallel with Message Passing Interface (MPI). The coprocessor works as a MPI node.

The major studies to accelerate Smith-Waterman algorithms includes XSW [29], SWAPHI [28], LSDBS [9] and SWIMM [52], whereby the LSDBS is a consequent work of XSW.

Features of these works are listed in Table 4. In this table GCUPS (giga cell updates per second) is the standard performance measurement of Smith-Waterman algorithm. Inspired by the success of SWIPE on CPU platforms, all of these works use inter-sequence parallelism and score profile to achieve peak performance. SWAPHI also implemented intra-sequence parallelism to prove that the inter-sequence parallelism is better. However, as the cache system is not so abundant than that on CPUs, a cache miss problem with long query sequence is reported by XSW and SWIMM. SWIMM proposed an variant of score profile, which is called adaptive profile to solve the cache miss problem. The performance is not very satisfying. SWAPHI computes 8 cells along the subject sequence before switching to the next query residue, while the XSW only computes 4 cells. This method effectively reduce memory access by trading off register usage. The LSDBS proposed a multi-pass method to solve the problem. The major idea is to scan the query sequence in multiple passes in order to improve the data access locality. This method achieves the best performance on Xeon Phi.

**Table 4:** The major studies for accelerating Smith-Waterman algorithm on Xeon Phi.

Study	Work mode	Perf (1 Phi)	CPU	DB size restrict
XSW	Native	70 GCUPS	N/A	Phi memory
SWAPHI	Offload	62 GCUPS	N/A	System memory
SWIMM	Offload	45 GCPUS	Yes	System memory
LSDBS	Offload	72 GCUPS	Yes	Hard disk

## 5 Open Issues in Big Biological Data Analytic

### 5.1 High Performance Computing

HPC is defined to speedup particular applications for efficiency. HPC is a must for most biological data analytics tasks to tackle the challenges of large amount of data and long running time. Overall, we categorize various HPC techniques into three directions, which are algorithm improvement, architecture-aware optimization and workflow optimization.

#### 5.1.1 Algorithm Improvement

This is to reduce time complexity for a specific algorithm. For example, a short sequence alignment employing brute force search has the complexity of exponential time. However, modern alignment programs usually adopt advanced indexing techniques, such as hashing, suffix trees or even Bloom Filter, which can reduce the complexity significantly. On the other hand, some algorithms trade accuracy for time, such as the sequence search algorithms BLAST and Smith-Waterman. However, there is always a limit to improve the time complexity. On the other hard, researchers also notice that even with the same complexity, the performance may vary greatly on different architectures. This is because a program's characteristics (compute and memory access patterns) may or may not fit into a specific hardware architecture. Following this clue, a number of studies are conducted for architecture-aware optimization.

#### 5.1.2 Architecture Aware Optimization

This refers to performance optimization on a particular hardware platform for a given application. The general idea is to optimize the algorithm's compute and memory access patterns, such as reorganization of data layouts, to fit into the architecture features. Note that nowadays CPUs are no longer the only available computing processors. Researchers are also interested in emerging parallel architectures, such as GPUs, Xeon Phi coprocessors, and FPGAs.

There are two major challenges when applying architecture-aware optimization techniques to biological data analytics algorithms. First, it is usually necessary to carefully tune or even redesign the algorithms to fit into the architecture features. For example, GPUs are suitable for massive data parallelism, but suffer seriously from irregular computation and memory access patterns. Unfortunately, many biological data analytics applications employ irregular data structures, such as the suffix tree index used by short read alignment, the sparse matrix used in SNP detection and the graph representation adopted in most genome assembly algorithms [78]. A lot of research efforts are taken to investigate and optimize those algorithms to make them suitable for the GPU architecture [5], [43], [53], [79], [80]. Another example is Xeon Phi, which features the 512-bit vector processing units (VPUs). Algorithms must be redesigned to take advantage from VPUs using intrinsics. Researchers have been working on particular optimization for biological algorithms on Xeon Phi, such as Smith-Waterman sequence alignment [28] and construction of whole-genome networks [81]. Second, those co-processors usually have their own limitations, which should be taken into account when designing algorithms. For example, they have very limited memory capacity, which is usually smaller than 10 GB. Typical applications, such as genome assembly, may consume hundreds of GB of memory, which is challenging to be implemented on such accelerators. Additionally, most accelerators communicate with CPUs via PCIe with the bandwidth of a few GB per second only. Therefore, data transfer between a host and accelerator must be minimized.

#### 5.1.3 Workflow Optimization

Besides the performance improvement and architecture aware optimizations, there is workflow optimization because of application dependency. The major purposes of workflow optimization are to facilitate the deployment on a distributed environment and reduce the overhead from data movement between individual programs. Researchers have been working on this direction for some typical workflows. For example, Crossbow [82] integrates sequence alignment (Bowtie [83]) and SNP detection (SOAPsnp [17]) into a single cloud-based solution. It combines and optimizes the two components in an automatic and parallel pipeline running on a single or multiple nodes. The similar workflow is also studied to eliminate the expensive external sorting between the sequence alignment and SNP detection [84].

### 5.2 Performance Scalability

For big biological data analytics applications, a single processor or accelerator usually cannot satisfy the performance requirement. As a result, researchers have been exploiting to scale biological applications to a large number of compute nodes in a cluster. Note that, in this section the scalability refers to the computing environment consisting of a number of processors that are not tightly coupled on the same chip. They may be either discrete processors within a server, such as CPUs and GPU, or distributed computing employing multiple compute nodes.

Some of biological data analytics applications are highly scalable to multiple nodes using task parallelism. To take short read alignment as an example, each node is usually able to hold the entire index data structure (typically around 2 GB for human genome), and then processes the reads assigned to this node. There is no dependency among different nodes. Crossbow [82] employs this solution to scale both sequence alignment and SNP detection in a cloud with multiple nodes.

Instead, some of biological data analytics applications are difficult to employ task parallelism because of dependency. Fine-grained data parallelism should be explored to scale the applications to a large number of nodes. One of the typical applications that belongs to this category is genome assembly [16]. Modern assembly algorithms are based on graph data structures and algorithms, such as graph construction, traversal and correction. Therefore, it suffers from most conventional issues for distributed graph processing, such as imbalanced workloads and heave communication overhead. There are many research efforts to try to address those issues on various hardware platforms [85], [86], [87], [88]. In general, better scalability can be achieved after careful algorithm redesign and tuning.

### 5.3 Programming Productivity

Biological data analytics also faces the challenge of programming productivity, which is similar to other HPC applications. Based on state-of-the-art HPC technologies, we discuss the programming productivity challenges from shared memory and distributed memory systems separately.

Traditional shared-memory parallel programming models mainly include POSIX Threads (Pthreads) and OpenMP. However, as many-core architectures are emerging recently, those programming models are either not well supported or unsuitable because of hardware's unique features. For example, GPUs adopt CUDA or OpenCL for programming. Xeon Phi can support OpenMP and Pthreads, but also encourages developers to use Intel Cilk Plus. Additionally, Xeon Phi has a set of 512-bit SIMD intrinsics to utilize VPUs, which essentially is the key of high performance on Xeon Phi. The advantage of using those programming languages that are offered by vendors is that they are capable of taking advantage of architecture-aware optimizations to fully utilize hardware resources. However, the disadvantages are the difficulty of programming and poor portability. Because of this reason, both research and industry are exploiting portable and efficient programming models for various many-core architectures. Fortunately, we have witnessed that efforts such as OpenCL and OpenACC have shed some light on heterogeneous computing. Additionally, researchers also port the MapReduce programming framework [89], which is originally proposed for distributed computing, to many-core architectures (such as to GPUs and Xeon Phi) to facilitate the parallel programming. However, both the portable programming frameworks (such as OpenCL) and MapReduce models sacrifice performance to ease the burden of parallel programming. For example, OpenCL has very limited capability to utilize SIMD VPUs on Xeon Phi. MapReduce is only suitable for data parallelism. Therefore, most developers today are still using vendor-offered programming languages to develop biological data analytics applications on shared memory systems for efficiency.

On the other hand, MPI is the most widely used programming model for distributed computing. Researchers utilize MPI to develop high-performance biological data analytics tools on supercomputers [85], [90]. However, due to the demanding requirements of scalability and fault tolerance, new programming models are proposed for large scale distributed computing, such as MapReduce and Spark. Those distributed programming frameworks improve the scalability as well as simplify the programming. Therefore, there are studies to deploy biological data analytics applications in cloud based on MapReduce [82], [91], [92]. However, data structures of some biological applications, such as the graph representation in genome assembly, do not naturally fit into the MapReduce's data parallelism model. Future research efforts to explore distributed graph processing frameworks (such as Pregel [93]) for such applications are worthwhile.

## 6 Conclusion

We have presented a survey of computing platforms for big biological data analytics in this paper. We identity two high-level categories of biological data analytics problems: those for analyzing whole sequence data and those for analyzing NGS data. We have discussed the characteristics of these two categories of problems as well as appropriate computing platforms used to solve them. Challenges of designing efficient big biological data analytics algorithms have also been listed. In addition, a case study that compares the performance of HPC Smith-Waterman algorithms on different computing platforms has been provided. Finally, we have added a discussion of open issues in designing HPC big biological data analytics algorithms.

## Conflict of interest

The authors declare that they have no conflict of interest.
